# Transcriptome Profiling and Chlorophyll Metabolic Pathway Analysis Reveal the Response of *Nitraria tangutorum* to Increased Nitrogen

**DOI:** 10.3390/plants12040895

**Published:** 2023-02-16

**Authors:** Chenggong Liu, Na Duan, Xiaona Chen, Xu Li, Naqi Zhao, Wenxu Cao, Huiqing Li, Bo Liu, Fengsen Tan, Xiulian Zhao, Qinghe Li

**Affiliations:** 1 Research Institute of Forestry, Chinese Academy of Forestry, Beijing 100091, China; 2 Key Laboratory of Tree Breeding and Cultivation, National Forestry and Grassland Administration, Beijing 100091, China; 3 Experimental Center of Desert Forestry, Chinese Academy of Forestry, Dengkou 015200, China; 4 National Long-Term Scientific Research Base of Ulan Buh Desert Comprehensive Control, National Forestry and Grassland Administration, Dengkou 015200, China

**Keywords:** transcriptome analysis, nitrogen addition, *Nitraria tangutorum*, chlorophyll metabolism

## Abstract

To identify genes that respond to increased nitrogen and assess the involvement of the chlorophyll metabolic pathway and associated regulatory mechanisms in these responses, *Nitraria tangutorum* seedlings were subjected to four nitrogen concentrations (N0, N6, N36, and N60: 0, 6, 36, and 60 mmol·L^−1^ nitrogen, respectively). The *N. tangutorum* seedling leaf transcriptome was analyzed by high-throughput sequencing (Illumina HiSeq 4000), and 332,420 transcripts and 276,423 unigenes were identified. The numbers of differentially expressed genes (DEGs) were 4052 in N0 vs. N6, 6181 in N0 vs. N36, and 3937 in N0 vs. N60. Comparing N0 and N6, N0 and N36, and N0 and N60, we found 1101, 2222, and 1234 annotated DEGs in 113, 121, and 114 metabolic pathways, respectively, classified in the Kyoto Encyclopedia of Genes and Genomes database. Metabolic pathways with considerable accumulation were involved mainly in anthocyanin biosynthesis, carotenoid biosynthesis, porphyrin and chlorophyll metabolism, flavonoid biosynthesis, and amino acid metabolism. N36 increased *δ*-amino levulinic acid synthesis and upregulated expression of the magnesium chelatase H subunit, which promoted chlorophyll *a* synthesis. Hence, N36 stimulated chlorophyll synthesis rather than heme synthesis. These findings enrich our understanding of the *N. tangutorum* transcriptome and help us to research desert xerophytes’ responses to increased nitrogen in the future.

## 1. Introduction

Nitrogen (N) is an essential macronutrient and key signal throughout the plant life cycle [1,2]. In the genetic evolution of plants, the N element is one of the most considerable limiting factors for plant metabolism, as it is a constituent of a variety of many biomolecules, such as amino acids, proteins, chlorophylls, phytohormones, and nucleic acids [3,4,5,6]. N fertilization is known to increase plant yield and productivity [7,8,9,10]; however, over the years, it has also led to increased N fertilizer use by farmers and ranchers. Ecologically, the excessive application of fertilizer has disastrous effects, such as eutrophication [1,11,12], soil acidification [13,14], and air pollution [15,16], as well as changes in the structure and diversity of plant and soil microbials [17,18]. Conversely, N deficiency also affects the activities and processes of plant life by altering the levels of many amino acids and the biosynthesis of some carbohydrates [19,20]. For example, poplar plants reduce N absorption and restrict N metabolism levels under N-deficiency conditions [21,22].

To detect and respond to variations in a soil’s available N levels, plants have evolved a variety of environmental adaptation strategies, including morphological characteristics and physiological and biochemical mechanisms [23,24,25]. However, N uptake, transformation, recycling, and reuse by plants is a capricious and complex process involving multiple genes [12,26]. Previous studies have shown that a large number of genes in *Arabidopsis thaliana* play roles in growth and multiple metabolism pathways [27,28,29], and plant responses to N starvation or excess conditions are particularly pronounced [30,31]. Under N-limited conditions, hundreds of genes were significantly differentially expressed in herbaceous plants, such as *Oryza sativa* [32], *Lycopersicon esculentum* [33], *Triticum aestivum* [4], and *Zea mays* [34]. There are similar research reports on timber and economic tree species, such as *Populus* [3,21], *Pyrus pyrifolia* [35], and *Citrus reticulata* [36].

As is well known, chlorophyll and nitrogen are closely linked leaf traits that determine C_4_ plants photosynthesis and productivity [37]. N is a considerable part of chlorophyll, and the concentration of N in the environment always affects the content and metabolic synthesis process of chlorophyll in plant tissues. For example, the leaf chlorophyll content of *O. sativa* increased with higher N fertilizer and was saturated when the amount of N fertilizer became excessive [38]. Goto et al. [39] demonstrated that plants in N-deficient soils can improve their growth mechanism by enhancing stringent responses to reduce chloroplast metabolism. The GATA58 gene of *Glycine max* can participate in the regulation of chlorophyll biosynthesis, and this regulation also responds to nitrogen addition [40]. Moreover, Sheng et al. [41] found that ferrochelatase 2 (*BrFC 2*), a single base mutation (*dBrFC2*) involved in plant heme synthesis, can simultaneously improve the heme and chlorophyll content of *Brassica*. However, the identification of transcription factors involved in regulating chlorophyll biosynthesis is still limited to herbaceous plants and remains poor for N-treated woody plants.

In recent decades, with the emergence of the global N deposition phenomenon, the impact of atmosphere and soil N on global ecosystems and plant growth is becoming increasingly significant [42]. Existing studies have shown that the high N deposition area in China is gradually expanding from the southeast to the northwest desert area [43]. Compared with forest ecosystems, desert ecosystems are ecologically sensitive areas with low natural soil N content, and even a small N addition will bring considerable ecological effects [44]. Among these ecological effects, changes in N availability have potential impacts on plant growth, productivity, metabolic processes, and molecular regulatory mechanisms. However, the effects of additional N input on gene expression and molecular mechanisms in arid desert plants have been inadequately studied.

*Nitraria tangutorum*, a member of the genus *Nitraria* in the Zygophyllaceae family, is a typical dryland xerophyte commonly found in the desert areas of northern China [45,46]. As a unique dominant species, *N*. *tangutorum* has a well-developed underground root system, numerous aboveground branches, and fleshy lobules [47]. These morphological characteristics not only provide congenital conditions for its growth, reproduction, and survival but also play a critical role in strengthening delicate ecosystems in desert regions, such as stabilizing moving dunes and reducing wind speed [48,49]. Likewise, *N*. *tangutorum* have considerable market value; for example, their fruits are used to make juices and medicines [50], and their dried branches and fallen leaves are key sources of firewood for nearby settlements [47]. Because of these important ecological and economic values, increasingly more attention has been paid to the research of *N*. *tangutorum*. For example, abiotic stresses, such as precipitation [46], drought [47], soil nutrients [51], and salt stress [52], will all affect the growth and development of *N. tangutorum*. Our previous study also found that the addition of 36 mmol·L^−1^ N fertilizer could promote the bud and branch formation of of *N*. *tangutorum* seedlings [53]; in addition, its chlorophyll content and fluorescence activity of leaves were related to the addition of N and P [54]. However, the molecular mechanisms by which *N. tangutorum* responds to nitrogen addition remains poorly studied. Therefore, in this article, we choose the ideal desert plant, *N. tangutorum*, as our research material. Our main goal is to share basic information regarding changes in the transcriptome of *N. tangutorum*, as well as the possible mechanisms that regulate its response to increased levels of N. The information obtained herein may contribute to the exploitation and conservation of various xerophytes.

## 2. Results

### 2.1. Growth and Biomass Affected by Nitrogen Treatment

**Figure 1:**
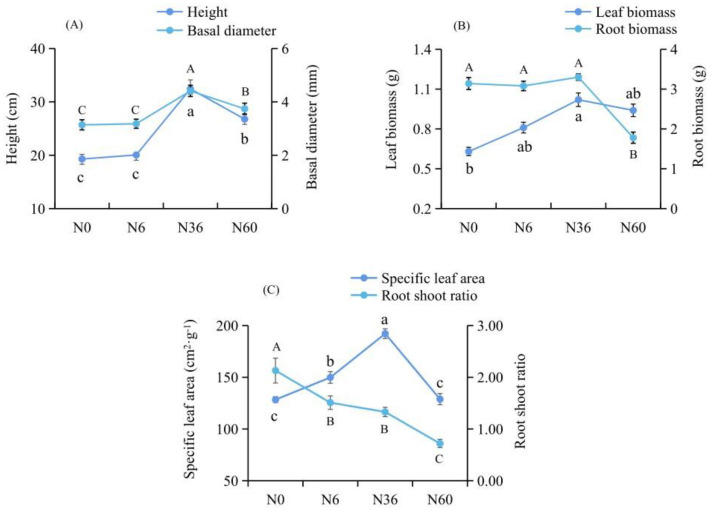
Growth of *N. tangutorum* treated with different N additions. (**A**) The height and basal diameter growth, (**B**) leaves and roots biomass, and (**C**) specific leaf area and root–shoot ratio of *N. tangutorum* treated with different N additions. Four N addition treatment levels (0 (N0), 6 (N6), 36 (N36), and 60 (N60) mmol·L^−1^) were established in the experiment, with N0 used as the control group. Results are expressed as means ± standard deviation based on three independent experiments. Broken lines with different letters indicate a significant difference (*p* < 0.05) as determined by analysis of variance and Duncan’s multiple range test.

Growth characteristics of *N. tangutorum* showed differences among the N concentration treatment groups (Figure 1). Compared with the control group (N0), the N6 treatment group had no significant effect on height, basal diameter, leaf biomass, or root biomass of *N. tangutorum* seedlings. The N36 group showed the highest height, basal diameter, leaf and root biomass, and specific leaf area, their averages being 32.44 ± 1.68 cm, 4.41 ± 0.21 mm, 1.02 ± 0.02 g, 3.30 ± 0.08 g, and 192.18 ± 4.69 cm^2^·g^−1^, respectively. When the N concentration was 60 mmol·L^−1^, the root biomass was significantly lower than that of the N0 group, and the biomass was only 56.69% of the N0 group. In addition, the root–shoot ratio of *N. tangutorum* seedlings decreased gradually with the increase of nitrogen concentration and was significantly lower than that of the N0 group. These results indicated that N36 could be considered as optimal N content for *N. tangutorum* seedlings growth, N60 as exceeding and depressing.

### 2.2. RNA-Seq Analysis and Transcript Splicing

Table S1 shows the raw reads of *N. tangutorum* RNA-seq, ranging from 43,516,318 (N36-1) to 57,280,550 (N6-3) under different N addition conditions. The ratio of clean reads to raw reads in each group ranged from 97.10% (N60-2) to 98.49% (N36-3), and the overall average clean reads rate reached 97.93%. These high ratios ensure the splicing of transcripts. Furthermore, the GC content of different treatment ranged from 45.44% (N36-3) to 46.41% (N6-3), with an error rate of 0.03%, which also met our study requirements (see Supplementary Data Table S1).

**Figure 2:**
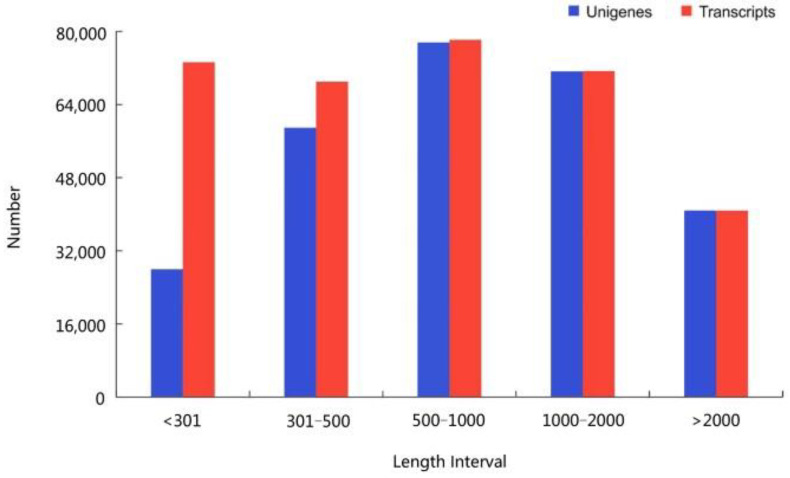
Length distributions of unigenes and transcripts.

We combined transcript sequences followed by performing hierarchical clustering to obtain 332,420 transcripts and 276,423 unigenes (Figure 2). We found 86,862 transcripts (31.42% of the total) that were 200–500 bp in length, 77,546 cDNA transcripts that were 500 bp–1 kbp long (28.05% of the total), 71,253 transcripts (25.78% of the total) with a length of 1–2 kbp, and 40,762 transcripts (14.75% of the total) with a length of >2 kbp. In summary, the sequencing and splicing results are of high quality and can be used for the subsequent analysis of gene expression levels.

### 2.3. GO Functional Annotation

**Figure 3:**
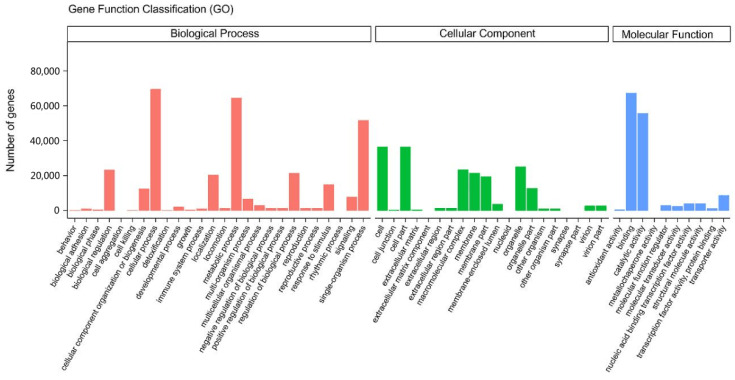
Gene ontology functional classifications.

The obtained transcript information was compared with the GO database, and the Blast2GO v2.5 software was used to determine the GO classification on all the assembled *N*. *tangutorum* unigenes. We then identified 122,945 genes that were functionally annotated before subdividing them into 56 GO functional groups. The annotated genes in the functional groups were classified according to the three major GO categories (biological process, cellular component, and molecular function), and the number of functional groups in each major category was 26, 20, and 10, respectively (Figure 3).

### 2.4. KOG Functional Classification

**Figure 4:**
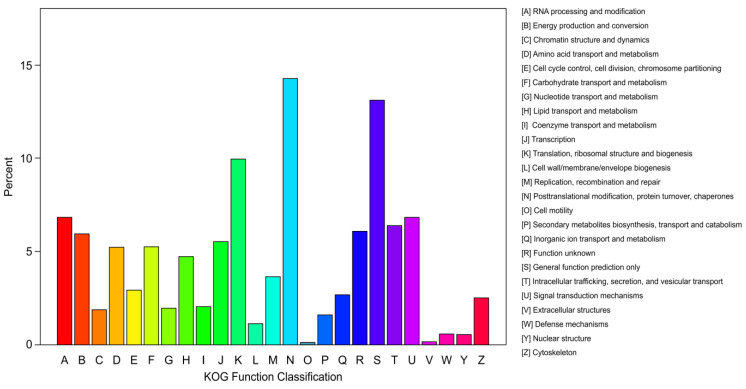
KOG functional classifications of *N. tangutorum* sample.

Comparing the transcript data with information in the KOG database showed that 48,287 unigenes (17.46%) were successfully annotated in the KOG database. According to the KOG functional classification, these genes were arranged into 26 groups (Figure 4). The five groups with the most unigenes, in order of prevalence, were post-translational modification, protein turnover, and chaperones; general functional genes only; translation, ribosome structure, and biogenesis; signal transduction mechanisms; and RNA processing and modification. These contained 6887 (12.8%), 6317 (11.7%), 4801 (8.9%), 3282 (6.1%), and 3273 (6.1%) unigenes, respectively. In addition, 3068 unigenes were related to intracellular transport, secretion, and vesicle control, and 2919 were classified as unknown.

### 2.5. DEGs Analysis

**Figure 5:**
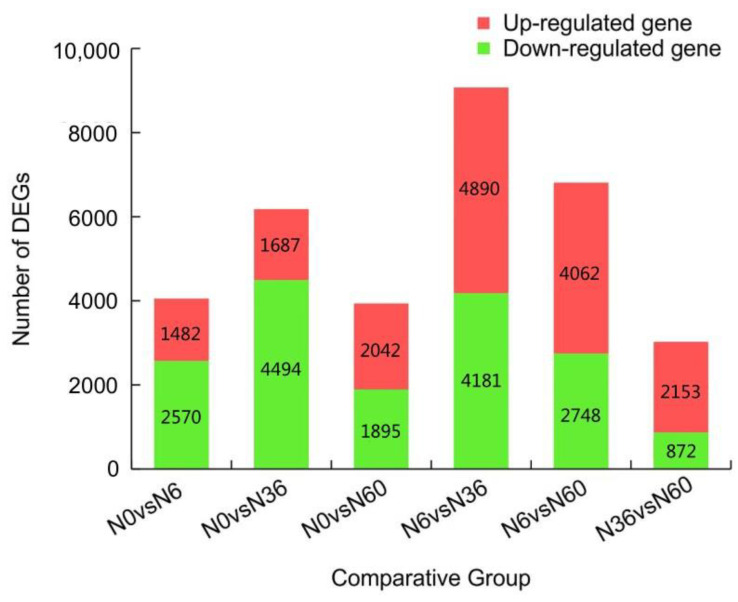
Numbers of differentially expressed genes obtained for each pairwise comparison.

Figure 5 shows the differences in gene expression among samples treated with different N concentrations. Compared with the N0 group, the number of DEGs in the N6-N36 group grew with the increase of N, and the numbers of upregulated and downregulated genes both expanded with increased N. However, the increase of the number of downregulated genes was significantly larger than that of upregulated genes. In the N60 group, the total number of DEGs was the least; however, the number of upregulated genes was significantly higher than the number of downregulated genes. In addition, principal component analysis (PCA) showed a clear cluster separation of the control (N0) and N treatment (N6, N36, and N60) groups (see Supplementary Data Figure S1).

**Figure 6:**
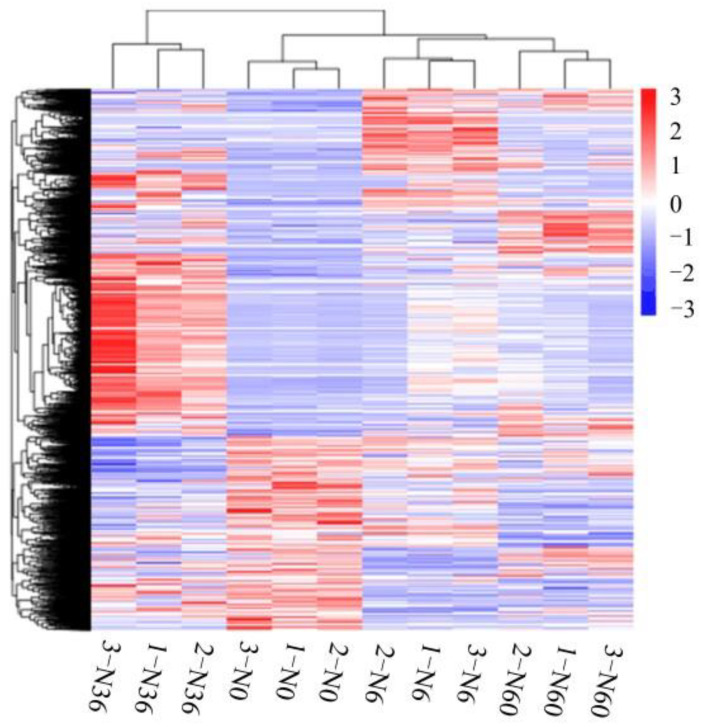
Cluster analysis of DEGs in *N. tangutorum* with different N additions. Color depths on the heatmap represent relative gene expression levels; log-transformed expression values ranging from -3 to 3; red indicates upregulated transcripts; blue indicates downregulated transcripts. 1-N0, 2-N0, and 3-N0: three replications of N0 treatment; 1-N6, 2-N6, and 3-N6: three replications of N6 treatment; 1-N36, 2-N36, and 3-N36: three replications of N36 treatment; and 1-N60, 2-N60, and 3-N60: three replications of N60 treatment.

We used cluster analysis to group similar genes, analyzed the functions of the previously known genes, and predicted those of the previously unknown genes (Figure 6). Genes clustered in the same group have similar functions and, in some cases, similar expression patterns; they may even participate in the same metabolic processes. As shown in Figure 6, the profiles of gene expression in N6 and N60 were more similar, and they can cluster into the same branch as N0.

### 2.6. Enrichment Analysis of DEGs in KEGG Pathways

The multiple signal transduction and metabolic pathways associated with *N. tangutorum* DEGs under different N additions were determined through KEGG enrichment analysis. We found that 1101 DEGs (see Supplementary File S1), representing 113 metabolic pathways in the N0 and N6 groups, were annotated in the KEGG database, among which 14 pathways showed significant enrichment (*p*-value < 0.05), such as ribosome, glycolysis/gluconeogenesis, and RNA degradation, and so on (Table 1); 2222 DEGs (see Supplementary File S1) were annotated in the KEGG database, representing 121 metabolic pathways in the N0 and N36 groups, and 10 pathways, including porphyrin and chlorophyll metabolism, were significantly enriched. In the comparison between N0 and N60 treatments, 1234 DEGs (see Supplementary File S1) were annotated to 114 metabolic pathways, involving 12 significantly enriched pathways, such as photosynthesis–antenna protein, chlorophyll metabolism, and zeatin synthesis. The significantly enriched KEGG pathways in the other comparison groups are detailed and shown in Table 1.

### 2.7. Metabolic Responses of Porphyrin and Chlorophyll to Increased Nitrogen

**Figure 7:**
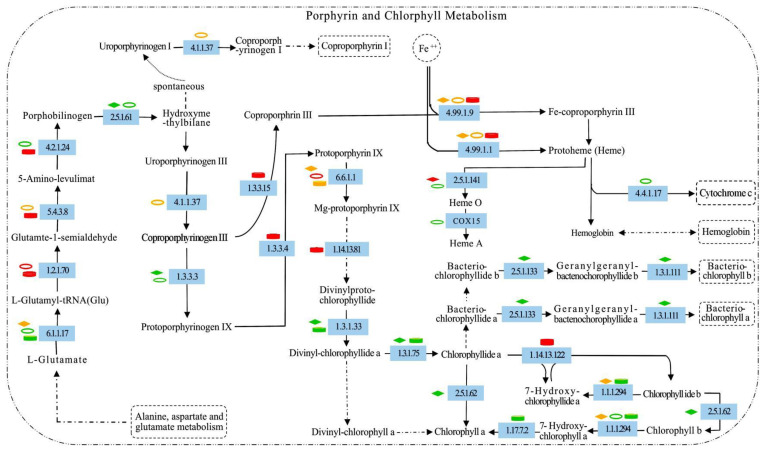
Porphyrin and chlorophyll metabolism pathways of *N. tangutorum* under different N treatments. (1) The diamond, concentric circle, and cylindrical shapes in the pathway maps represent the experimental treatments N6, N36, and N60, respectively; red shapes indicate the upregulated genes; green shapes indicate the downregulated genes; and yellow shapes indicate the up/downregulated genes. (2) Black solid arrows denote molecular interactions or relationships; dotted arrows denote indirect effects; and the beginning or ending of the signal pathway is shown as a dotted boxes. (3) Genes or enzymes involved in metabolic pathways are shown on a blue background. 6.1.1.17: glutamine tRNA synthetase; 1.2.1.70: glutamyl-tRNA reductase; 5.4.3.8: glutamate-1-hemialdehyde transaminase; 4.2.1.24: bile pigment synthase; 2.5.1.61: bile pigment deaminase; 4.1.1.37: uroporphyrinogen decarboxylase; 1.3.3.3: coproporphyrinogen III oxidase; 1.3.3.4 and 1.3.3.15: protoporphyrinogen III oxidase; 4.99.1.1 and 4.99.1.9: ferrochelatase; 2.5.1.141: heme O ferric synthetase; 4.4.1.17: cytochrome c heme-lyase; 6.6.1.1: magnesium chelatase H subgroup; 1.14.13.81: magnesium porphyrin; 1.3.1.33: protochlorophyllide reductase; 1.3.1.75: divinyl reductase; 2.5.1.62: chlorophyll synthase; 1.14.13.122: chlorophyllinate a oxidase; 1.1.1.294: chlorophyll b reductase; 1.3.7.12: red chlorophyll catabolite reductase; 2.5.1.133: chlorophyll synthase; 1.3.1.111: CHIP; and 1.17.7.2: 7-hydroxymethyl chlorophyll a reductase.

Figure 7 showed that the activation of metabolic pathways related to porphyrin and chlorophyll synthesis varied with N concentration. In the ALA synthesis pathway, glutamyl-tRNA synthase (6.1.1.17) gene expression was downregulated in N6, while glutamyl-tRNA reductase (1.2.1.70) gene expression levels remained unchanged. The difference was that the gene expression of tRNA synthase was downregulated in N36, while the gene expression of glutamine-tRNA reductase was upregulated relative to N6, and glutamine-1-hemialdehyde transaminase showed a mixed trend of up/downregulation. In N60, tRNA synthase gene expression was downregulated, glutamine-tRNA gene reductase and glutamine-1-hemialdehyde transaminase (5.4.3.8) gene expression were upregulated, and the ALA synthesis gene was also upregulated. In the protoporphyrin IX (ProIX) synthesis pathway, the gene-expression levels of bile pigment deaminase (2.5.1.61) downregulated in N6 and N36; the coproporphyrinogen III oxidase (1.3.3.3) gene had a downregulated expression in N6; the bile pigment synthase (4.2.1.24) gene had a downregulated expression in N36; and the gene expression of uroporphyrinogen decarboxylase (4.1.1.37) was variable up/downregulated. In another pathway related to protoporphyrin IX synthesis, the expression of uroporphyrinogen decarboxylase was up/downregulated to varying degrees; the coproporphyrinogen III oxidase (1.3.3.3) gene had a downregulated expression; and the gene expressions of bile pigment synthase (4.2.1.24) and protoporphyrinogen III oxidase (1.3.3.4 and 1.3.3.15) were upregulated in N60.

With protoporphyrin IX as the branch, two metabolic pathways were formed, namely the chlorophyll and haem synthesis pathways. In N6, expression changes in the ferrochelatase (4.99.1.1 and 4.99.1.9) gene were up/downregulated, while the ferrous heme O synthetase (2.5.1.141) gene was upregulated. In another chlorophyll synthesis pathway, the expression levels of the magnesium-chelating enzyme H subunit (6.6.1.1), chlorophyll acid ester and divinyl reductase (1.3.1.33 and 1.3.1.75), and chlorophyll synthetase (2.5.1.62) were reduced, and the chlorophyll *b* reductase (1.1.1.294) gene was downregulated in expression. In N36, the expression of the ferrochelatase (4.99.1.1 and 4.99.1.9) gene was up/downregulated. However, compared with N6, the expression of ferrous heme O synthetase (2.5.1.141) and COX15 was downregulated in N36, as was ferrous heme O and ferrous heme A synthesis. Cytochrome *c* heme-lyase (4.4.1.17) gene expression was downregulated, while the magnesium chelatase H subunit (6.6.1.1) gene expression was upregulated, and the downstream chlorophyll *b* reductase (1.1.1.294) gene expression was downregulated. In N60, the expression of the ferrochelatase (4.99.1.1 and 4.99.1.9) gene and the magnesium protoporphyrin (1.14.13.81) gene was upregulated, while the magnesium chelatase H subunit gene expression was up/downregulated. Compared with N36, the expression levels of protochlorophyllide reductase (1.3.1.33) and diethylene reductase (1.3.1.75) genes were downregulated in N60. The expression of the chlorophyll *a* oxidase (1.14.13.122) gene was upregulated, and the expression levels of chlorophyll and 7-hydroxymethyl chlorophyll *a* reductase (1.1.1.294 and 1.17.7.2) were reduced. In N6, the expression of red chlorophyll catabolite reductase (1.3.7.12) and the production of the red chlorophyll-degradation product formed during magnesium removal and transplant-based reactions were reduced, and the synthesis of the primary fluorescence chlorophyll-degradation product was inhibited. In addition, the expression levels of chlorophyll synthase (2.5.1.133) and CHIP (1.3.1.111) were downregulated.

### 2.8. Transcriptome Data Verification

**Figure 8:**
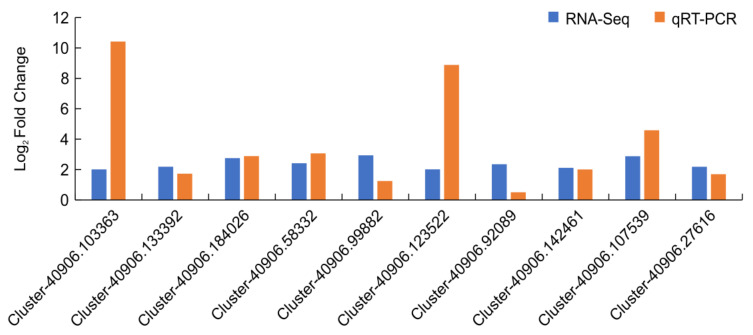
Results validation of *N. tangutorum* RNA-seq data under different N treatments.

We selected 10 DEGs that were highly related to N treatments for qRT-PCR analysis. Figure 8 shows that the RNA-Seq data and expression trends were similar, corroborating the accuracy of the RNA-Seq results, although there were differences in the absolute fold changes between the two methods.

## 3. Discussion

*Nitraria tangutorum* is the predominant plant class in the Ulan Buh Desert [46,55,56], and its leaves had a high storage capacity for C and N [51]. Previous studies have shown that growth and biomass production of plant was accelerated by N application [57] but was inhibited when the N supply was excessive [24]. Similarly, our study showed that the height, basal diameter, specific leaf area, and leaf biomass of *Nitraria tangutorum* seedlings were significantly increased by N36, but root biomass and root shoot ratio were inhibited in N60. That is, N36 could be considered as optimal N content for *N. tangutorum* seedlings growth but more likely to be inhibited under high N concentrations. Moreover, previous studies have shown that nitrogen supplementation not only affects the growth phenotype of plants but also leads to differential expression of their genes. Thus, it is of considerable value to explore its gene expression mechanisms in response to increased N under the context of phenotypic differences. In this paper, we studied the transcriptome of *N. tangutorum* in response to different N additions and established an 89.67 Gb *N. tangutorum* transcriptome database in which the proportion of clean reads to raw reads reached 97.93%, indicating high sequencing quality [49]. Our study also found that the N36 treatment produced the highest number of clean reads, possibly because N36 induced the diversified expressions of numerous *N. tangutorum* genes. In addition, more transcripts and unigenes were found in this study of *N. tangutorum* than in a previous study of *Nitraria sibirica* under salt stress [58], indicating that *N. tangutorum* has its own genetic differences in response to different environmental stresses.

In general, plant gene expression is unavoidably affected by a variety of environmental stressors during its growth and development [59,60]. When N availability fluctuates, plants take a number of steps to cope with the new environment [25,61]. For instance, a large number of *Populus tomentosa* genes were annotated into multiple KEGG pathways, among which multiple pathways related to amino acid and carbohydrate metabolism were significantly enriched, indicating that *P. tomentosa* shows a significant response to low N stress [20]. In our study, transcriptome data revealed that multiple genes in the leaves of *N. tangutorum* were differentially expressed under N addition, which was also similar to a previous study that found that the expressions of most ammonia transporter genes in poplar plants were significantly upregulated under low N stress [22]. KEGG metabolic-pathway-enrichment analysis for DEGs showed that the porphyrin/chlorophyll metabolic pathways were significantly enriched following each treatment; thus, we speculated that these responses may be associated with the diversity of enzymes (e.g., GS) in plants, reflecting the complexity of their roles in plants growth [24,62]. Other studies have shown that the plant “ribosome” pathway has undergone considerable changes under abiotic stress (e.g., drought stress [63]). A similar phenomenon was reflected in this study; the ribosome pathway was the KEGG pathway with the most significant difference between N0 and N6, indicating that a small N addition was more conductive to the differential expression of *N*. *tangutorum* ribosomal-pathway-related genes. Moreover, Anthocyanins are natural colorants belonging to the flavonoid family that have been shown to possess potent antioxidant properties [64]. Wang et al. [52] found that the “lavonoid synthesis” pathway was significantly enriched, and an anthocyanin synthesis gene, Oxoglutarate/iron-dependent dioxygenase (2-GO), was annotated by GO and highly upregulated, and similar to our study, they also found the anthocyanin synthesis, flavonoid biosynthesis, photosynthetic–antenna protein, amino acid biosynthesis, and metabolism processes were also significantly enriched. Thus, we speculate that upregulation of anthocyanin-related genes indicated that anthocyanins play an important role in reactive oxide species scavenging in *N. tangutorum* under N addition. In a word, the addition of N leads to changes in the external growth environment of *N. tangutorum*, which may make *N. tangutorum* more sensitive to external environmental stress and ultimately lead to a more frequent adjustment of transcriptional output [65].

Chlorophyll is the key factor involved in plant photosynthesis, providing energy for plant growth, development, and productivity [66,67,68]. As a component of the chloroplasts, increased nitrogen is beneficial for chlorophyll synthesis, up to a point [69,70,71]. As is well known, the molecular regulation of chlorophyll biosynthesis is a complex process, affected by the external abiotic factor and regulated by related genes [72,73]. The first step in chlorophyll biosynthesis is ALA synthesis, which is synthesized from glutamic acid tRNA synthetase, *δ*-ketoglutaric acid tRNA synthetase, GluTR tRNA reductase, and GSA-AT. In this paper, our data analysis showed that compared with the control, GluTR was not expressed in N6 but induced in N36 and was further upregulated in N60. With the increase in N addition, the expression trend of glutamyl tRNA reductase was consistent with glutamine-1-hemialdehyde transaminase. These results were not completely consistent with the expression of genes related to chlorophyll biosynthesis in maize leaves under zinc stress [74], indicating that different abiotic stresses had different effects on chlorophyll synthesis in plants. Thus, we predicted that the expression levels of these two enzyme genes in *N. tangutorum* would increase with increased N addition. Previous reports have shown that the expression of ALA-synthesis-related genes can affect the chlorophyll contents of plants [41] and that GluTR can regulate ALA synthesis at the transcriptional level, thereby affecting chlorophyll synthesis [75,76]. Here, we obtained similar results and speculated that glutamyl tRNA reductase is likely to be a key regulatory site for ALA synthesis when *N. tangutorum* receive external N input.

The second considerable pathway for chlorophyll synthesis involves the synthesis of protoporphyrin IX [77]. This pathway begins with ALA, from which protoporphyrin IX is formed through isomerization, decarboxylation, and oxidation reactions catalyzed by bile pigment synthase, bile pigment deaminase, uroporphyrin proporphyrin III synthase, uroporphyrin proporphyrin III decarboxylase, coproporphyrin III oxidase, and protoporphyrinogen III oxidase. In the present study, the bile pigment synthase gene was not expressed in N6 but was low-expressed in N36 and overexpressed in N60, indicating that the N concentration in the environment determined the bile pigment synthase expression level in *N*. *tangutorum* leaves. In addition, the gene-encoding bile chromatogen deaminase was downregulated in N6 and N36 but not expressed in N60. Both up/downregulation of the uroporphyrin proporphyrin III decarboxylase gene were observed in N36; however, there was more downregulation than upregulation. Therefore, we speculate that upregulation of the bile pigment deaminase and uroporphyrin proporphyrin III decarboxylase genes could occur at appropriate levels following N addition. However, the specific regulation mode of these differentially expressed genes remains to be further studied.

The coproporphyrin III oxidase gene was downregulated in N6 and N36, and ProIX oxidase was upregulated in N60; thus, it is possible that these two genes coordinate with each other before ProIX synthesis to increase ProIX expression under high N concentration. When ProIX production is catalyzed by ferrochelatase, it forms ferrous heme and then enters the heme synthesis pathway; when ProIX encounters magnesium chelase, Mg-protoporphyrin IX enters the chlorophyll synthesis pathway [78]. Previous findings showed that plants controlled the flow of ProIX towards chlorophyll synthesis by regulating magnesium chelase [79]. Similarly, the affinity of iron chelase for ProIX is lower than that of magnesium chelase, indicating that most ProIX leads to chlorophyll synthesis [80]. Furthermore, these genes were involved mainly in the chlorophyll synthesis pathway in the three experimental groups, and the expression of genes related to heme synthesis was very low. Compared with the controlled plants, the magnesium chelatase H subgroup gene of the N6 group was downregulated. Under increasing N, the upregulated expression of this gene was observed in N36, and although both up/downregulation of this gene were observed in N60, it was less upregulated than in N36, which led us to speculate that the expression of a magnesium-chelating enzyme during N processing is beneficial for chlorophyll synthesis over heme synthesis. Similarly, Liu et al. [41] found in the study of *Brassica* that the CAB domain in *BrFC2* was not the structure to maintain the catalytic activity of heme enzyme, and only after the single base mutation of *dBrFC2* could both increase the content of chlorophyll and heme in plants. It can be seen that N36 treatment is likely to change the domain of one or more genes in the chlorophyll synthesis of *N*. *tangutorum* but did not cause mutations in genes related to heme synthesis. Previously, it was shown that chlorophyllin a synthesis was catalyzed by magnesium chelase and other enzymes, such as POR [81]. Here, we found that the expression of protochlorophyllide reductase was lower in both N6 and N60 and that the degree of downregulation was almost the same in each case, whereas the enzyme was not expressed in N36. These results indicated that original chlorophyll ester reductase expression affected chlorophyll ester *a* synthesis by reducing its expression at both low and high nitrogen concentrations. In addition, the translation of chlorophyll *b* into chlorophyll *a* is likely to be part of the chlorophyll degradation pathway [82]. In the present study, relative to the control, we found that chlorophyll *b* reductase gene expression was lower in N6, N36, and N60, and the capability to translate chlorophyll *b* to chlorophyll *a* was reduced at higher N concentrations. Thus, we speculated that increased nitrogen addition could reduce chlorophyll degradation.

## 4. Materials and Methods

### 4.1. Plant Growth Conditions and Experimental Treatments

*N. tangutorum* plants in the Ulan Buh desert (Inner Mongolia Dengkou, China; 106°45′ E, 40°26′ N) were selected as the research object in our study. The *N. tangutorum* seedlings used in the experiment were all cultivated using seeds taken from an adult plant that lives independently in the wild. On 13 March 2015, seedlings began to be bred in nutrient pots in the greenhouse of the Experimental Center of Desert Forestry, Chinese Academy of Forestry (106°43′ E, 40°24′ N) [47]. To reduce the impact of the experimental seedling growth difference on the research results, uniformly growing seedlings were selected from the above-bred half-sib families, then transplanted into self-developed PVC barrels (40 cm height × 16 cm in diameter) on 8 May 2015, with one plant per barrel. The soil matrix (the main physicochemical properties of the soil matrix are given in Table 2) consisted of local abandoned cornfield topsoil and river, which was sand mixed 1:1 (*v*/*v*) and screened. On 23 May 2015 (15 days after transplanting), the seedlings in the PVC barrels were treated with N fertilizer (N content 46.7% of urea (Shaanhua Coal Chemical Industry Group Co., Ltd., Shanxi, China)). Four additional N treatment levels (0 (N0), 6 (N6), 36 (N36), and 60 (N60) mmol·L^−1^) were established in the experiment, each with 25 replicates per treatment, with N0 used as the control group. To prevent the effects of natural precipitation on the experimental results, a transparent canopy was built and used to control the water supply. On 1 August 2015 (70 days after N treatment), leaf samples were collected from each treated seedling between 9:30 and 10:30 am. We randomly selected 18 barrels of seedlings from each treatment, 6 mature leaves were collected from each seedling, and a total of 108 leaves were collected for each treatment. We divided the 6 leaves of each seedling into 3 two-leaf repetitions. Each treatment therefore had three repetitions with 36 mixed leaves per repetition. Then, the samples were quickly stored at −80 °C until use. In addition, we measured and counted the plant height, basal diameter, specific leaf area, leaf biomass, root biomass and root–shoot ratio of *N. tangutorum* seedlings with different N treatments before sampling.

### 4.2. Library Preparation for Transcriptome Sequencing

Total RNA of *N. tangutorum* was isolated from their leaf powder (three biological plants replicated per treatment) using the RNAsimple Total RNA Kit (Tiangen, Beijing, China). Then, 1.5 µg RNA was taken from each sample as the input material for the RNA preparations. Sequencing libraries were generated according to Liu et al.’s method [47]. The mRNA was isolated and purified from the total RNA using poly-toligo-attached magnetic beads. Next, first-strand cDNA was synthesized using reverse transcriptase, and second-strand cDNA was synthesized using DNA polymerase I and RNase H (Novogene Technology Co., Ltd., Beijing, China). The library fragments were purified using the AMPure XP system (Beckman Coulter, Beverly, MA, USA) to obtain the target cDNA fragments. Then, 3.0 µL USER Enzyme (NEB, Ipswich, MA, USA) was used with size-selected, adaptor-ligated cDNA at 37 °C for 15 min followed by 5 min at 95 °C before PCR. Next, PCR was performed with Phusion High-Fidelity DNA polymerase, Universal PCR primer, and Index (X) Primer. Library quality was evaluated on the Agilent Bioanalyzer 2100 system (Agilent Technologies, Santa Clara, CA, USA) and, finally, PCR amplification was performed to obtain the complete cDNA library. All of these experiments were performed with the help of Novogene Technology Co., Ltd. (Beijing, China).

### 4.3. Enrichment Analysis of Differentially Expressed Genes

The analysis and screening of the differentially expressed genes (DEGs) were performed using the DESeq software [83]. The numbers of DEGs between the N0 groups and N addition treatment groups were counted, including the up/downregulated genes. The *p*-value was adjusted using the Benjamini and Hochberg method. The corrected *p*-value of <0.01 and a log2-fold change > 1 were set as the threshold for significant differential expression [47]. ImageGP (http://www.ehbio.com/ImageGP, accessed on 16 October 2015) was used to achieve the GO analyses [29]. All the obtained DEG sequences were annotated in the Kyoto Encyclopedia of Genes and Genomes (KEGG) database by KOBAS (2.0) (http://kobas.cbi.pku.edu.cn/, accessed on 23 November 2015) to identify the signal transduction and metabolic pathways involved in DEGs. When the *p*-value threshold of ≤0.05 was obtained, the KEGG analysis was considered significantly enriched by DEGs [84]. All raw data obtained from the experiments described above are available for review in Supplementary File S2.

### 4.4. Verification of RNA Sequencing Data by RT-qPCR

To verify the RNA-Sequencing data, 10 candidate DEGs (1, 7, and 2 DEGs from the N0 vs. N6, N0 vs. N36, and N0 vs. N60 comparison groups, respectively) of *N*. *tangutorum* were randomly selected, and qRT-PCR analysis was conducted to validate the differences in their expression levels. The specific primers were designed using PRIMER5 software (http://www.PremierBiosoft.com, accessed on 17 January 2016) [85] and are listed in Supplementary Data Table S2. We reverse-transcribed 1.0 μg of the total RNA of each *N*. *tangutorum* sample with the Goldenstar^TM^ RT6 cDNA Synthesis Kit (Novogene Technology Co., Ltd., Beijing, China); then, the cDNA was amplificated using 2 × T5 Fast qPCR Mix (SYBR Green I). PCR amplification was performed under the following conditions: one cycle of 1 min at 95 °C, followed by 40 cycles at 95 °C for 10 s, 60 °C for 5 s, and 72 °C for 10 s. Afterward, the relative gene expression levels were analyzed using the 2^−ΔΔCt^ method [86]. The results were analyzed by using the Bio-Rad CFX Manager 3.1 software (Bio-Rad, Hercules, CA, USA). qRT-PCR for each *N*. *tangutorum* sample was repeated three times.

## 5. Conclusions

A total of 14,170 DEGs were identified. There were 1101, 2222, and 1234 DEGs assigned to the N0 vs. N6, N0 vs. N36, and N0 vs. N60 groups in the 113, 121, and 114 metabolic pathways classified in the KEGG database, respectively. The metabolic pathways upregulated by increased nitrogen included anthocyanin biosynthesis, carotenoid biosynthesis, porphyrin and chlorophyll metabolism, flavonoid biosynthesis, and amino acid metabolism. The magnesium chelatase H subunit was upregulated in N36, promoting chlorophyll a synthesis, implying that N36 metabolism favored chlorophyll synthesis over heme synthesis.

## Figures and Tables

**Table 1:** Enrichment analysis of DEGs in KEGG pathways in different N addition treatments vs. control group comparison.

Comparative Group	KEGG Pathway	ID	DEG Number	*p*-Value
N0 vs. N6	Ribosome	ko03010	72	1.35 × 10^−5^
	Anthocyanin biosynthesis	ko00942	7	0.000142
	Carotenoid biosynthesis	ko00906	13	0.001063
	Porphyrin and chlorophyll metabolism	ko00860	20	0.001957
	Flavonoid biosynthesis	ko00941	10	0.002196
	Taurine and hypotaurine metabolism	ko00430	7	0.010283
	Proteasome	ko03050	19	0.011698
	Amino sugar and nucleotide sugar metabolism	ko00520	27	0.012803
	Photosynthesis–antenna proteins	ko00196	13	0.013312
	Glutathione metabolism	ko00480	21	0.013332
	Glycolysis/Gluconeogenesis	ko00010	37	0.013506
	RNA degradation	ko03018	28	0.018797
	Arachidonic acid metabolism	ko00590	8	0.021011
	Inositol phosphate metabolism	ko00562	16	0.022433
N0 vs. N36	Ribosome	ko03010	138	7.15 × 10^−8^
	Citrate cycle (TCA cycle)	ko00020	39	0.000176
	Proteasome	ko03050	40	0.000208
	Oxidative phosphorylation	ko00190	60	0.001279
	Protein processing in endoplasmic reticulum	ko04141	95	0.002001
	Phagosome	ko04145	45	0.002573
	Spliceosome	ko03040	76	0.023464
	Porphyrin and chlorophyll metabolism	ko00860	28	0.034622
	Valine, leucine, and isoleucine biosynthesis	ko00290	10	0.039746
	Alanine, aspartate, and glutamate metabolism	ko00250	32	0.045639
N0 vs. N60	Starch and sucrose metabolism	ko00500	54	6.68 × 10^−5^
	Cutin, suberine, and wax biosynthesis	ko00073	13	0.000312
	Limonene and pinene degradation	ko00903	10	0.002202
	Pentose and glucuronate interconversions	ko00040	23	0.00236
	Porphyrin and chlorophyll metabolism	ko00860	19	0.013013
	Protein processing in endoplasmic reticulum	ko04141	52	0.021174
	Stilbenoid, diarylheptanoid, and gingerol biosynthesis	ko00945	8	0.022509
	Fatty acid degradation	ko00071	18	0.023562
	Photosynthesis–antenna proteins	ko00196	13	0.029707
	Zeatin biosynthesis	ko00908	5	0.030729
	Anthocyanin biosynthesis	ko00942	4	0.033286
	C5-branched dibasic acid metabolism	ko00660	4	0.036787
N6 vs. N36	Carbon fixation in photosynthetic organisms	ko00710	94	1.86 × 10^−6^
	Pentose phosphate pathway	ko00030	66	4.71 × 10^−6^
	Starch and sucrose metabolism	ko00500	121	8.61 × 10^−6^
	Glycerolipid metabolism	ko00561	57	1.86 × 10^−5^
	Carotenoid biosynthesis	ko00906	30	9.65 × 10^−5^
	Anthocyanin biosynthesis	ko00942	10	0.001761583
	Glycine, serine, and threonine metabolism	ko00260	54	0.003925873
	Ether lipid metabolism	ko00565	21	0.006187502
	Pyruvate metabolism	ko00620	63	0.006361691
	Tryptophan metabolism	ko00380	28	0.009916273
	Citrate cycle (TCA cycle)	ko00020	43	0.011383806
	Arginine and proline metabolism	ko00330	39	0.015053622
	Porphyrin and chlorophyll metabolism	ko00860	40	0.016717157
	Amino sugar and nucleotide sugar metabolism	ko00520	64	0.02212956
	Fructose and mannose metabolism	ko00051	52	0.026971638
	Lysine degradation	ko00310	25	0.027587367
	Pentose and glucuronate interconversions	ko00040	42	0.02856809
	Betalain biosynthesis	ko00965	3	0.041318758
	Nitrogen metabolism	ko00910	31	0.043273338
N6 vs. N60	Stilbenoid, diarylheptanoid, and gingerol biosynthesis	ko00945	19	2.10 × 10^−6^
	Flavonoid biosynthesis	ko00941	18	2.22 × 10^−5^
	Limonene and pinene degradation	ko00903	15	0.000202
	Monoterpenoid biosynthesis	ko00902	10	0.000382
	Anthocyanin biosynthesis	ko00942	8	0.000603
	Arachidonic acid metabolism	ko00590	15	0.000738
	Carotenoid biosynthesis	ko00906	17	0.002699
	Glutathione metabolism	ko00480	33	0.005837
	Cutin, suberine, and wax biosynthesis	ko00073	13	0.008832
	Lysine biosynthesis	ko00300	10	0.009018
	Ascorbate and aldarate metabolism	ko00053	25	0.009493
	ABC transporters	ko02010	14	0.009879
	Pentose and glucuronate interconversions	ko00040	28	0.012063
	Phenylpropanoid biosynthesis	ko00940	32	0.017049
	Starch and sucrose metabolism	ko00500	59	0.027008
	Fatty acid degradation	ko00071	24	0.03139
	Monobactam biosynthesis	ko00261	5	0.041373
	Taurine and hypotaurine metabolism	ko00430	8	0.044949
N36 vs. N60	Proteasome	ko03050	27	3.52 × 10^−5^
	Biosynthesis of unsaturated fatty acids	ko01040	13	0.001866
	Steroid biosynthesis	ko00100	15	0.005451
	Fatty acid elongation	ko00062	10	0.006054
	Ribosome	ko03010	60	0.01207
	Citrate cycle (TCA cycle)	ko00020	18	0.022708
	Oxidative phosphorylation	ko00190	30	0.025972
	Limonene and pinene degradation	ko00903	7	0.036772
	Sesquiterpenoid and triterpenoid biosynthesis	ko00909	8	0.038735

**Table 2:** Main physicochemical properties of soil matrix (mean ± standard deviation).

Soil Index	Mean	Soil Index	Mean
pH	6.15 ± 0.146	Organic carbon content (g·kg^−1^)	6.20 ± 0.200
Porosity (%)	18.42 ± 0.055	Total N content (g·kg^−1^)	2.08 ± 0.035
Water content (g·cm^−3^)	1.03 ± 0.035	Total P content (g·kg^−1^)	0.12 ± 0.036
Bulk density (%)	49.99 ± 0.335		

## Data Availability

The data presented in this study are available on request from the corresponding author. The data are not publicly available due to ethical reason.

## Supplementary Materials

The following are available online at https://www.mdpi.com/article/10.3390/plants12040895/s1, Figure S1: Principal component analysis (PCA) of control (N0) and N treatment (N6, N36, and N60) groups; Table S1: Statistical analysis of *Nitraria tangutorum* RNA-Seq data, following treatment with supplemental nitrogen; Table S2: Description of the 10 primers designed; File S1: DEGs KEGG pathway enrichment results; File S2: All DEGs (*p*-value and log2-fold change applied), read count matrix, KEGGs, GOs, and KOGs for each treatment group vs. control group.
