# Supplementary material for: Transcriptome Profiling and Chlorophyll Metabolic Pathway Analysis Reveal the Response of Nitraria tangutorum to Increased Nitrogen
Source: Plants (Basel). 2023 Feb 16;12(4):895. doi: 10.3390/plants12040895 (PMC9962214; doi:10.3390/plants12040895)
Supplement: Supplementary file 1 [file plants-12-00895-s001.zip › Supplementary Data.pdf]

Table S1. Statistical analysis of *Nitraria tangutorum* RNA-Seq data, following treatment with supplemental nitrogen. Raw reads (Rr): raw sequencing data. Clean reads (Cr): number of reads after culling low-quality reads, adaptors and ambiguous reads. Clean bases (Cb): number of sequenced sequences multiplied by its length, and converted to units of G. Error rate (Er): base error rate. Q20 and Q30: the ratio of bases with *Phred*-value > 20 and *Phred*-value 30 to the total number of bases, respectively. GC content: G and C ratio considering the all bases. N0-1, N0-2, N0-3: three replications of N0 treatment; N6-1, N6-2, N6-3: three replications of N6 treatment; N36-1, N36-2, N36-3: three replications of N36 treatment; N60-1, N60-2, N60-3: three replications of N60 treatment.

Sample	Rr	Cr	Cb (Gb)	Er (%)	Q20 (%)	Q30 (%)	GC (%)
N0-1	54,230,138	53,229,668	7.98	0.03	97.32	92.60	46.24
N0-2	48,422,264	47,233,114	7.08	0.03	96.64	91.16	46.00
N0-3	53,158,862	52,212,380	7.83	0.03	97.16	92.26	45.86
N6-1	48,142,380	47,149,054	7.07	0.03	96.82	91.51	46.11
N6-2	48,985,550	48,009,132	7.20	0.03	96.84	91.56	46.04
N6-3	57,280,550	56,119,784	8.42	0.03	97.11	92.21	46.41
N36-1	43,516,318	42,487,874	6.37	0.03	97.24	92.47	45.71
N36-2	54,822,682	53,847,652	8.08	0.03	96.77	91.47	45.92
N36-3	56,660,722	55,806,872	8.37	0.03	97.48	93.00	45.44
N60-1	50,239,526	49,202,402	7.38	0.03	96.98	91.87	45.99
N60-2	45,061,990	43,756,516	6.56	0.03	97.12	92.20	45.92
N60-3	50,042,994	48,853,114	7.33	0.03	97.55	93.15	45.91

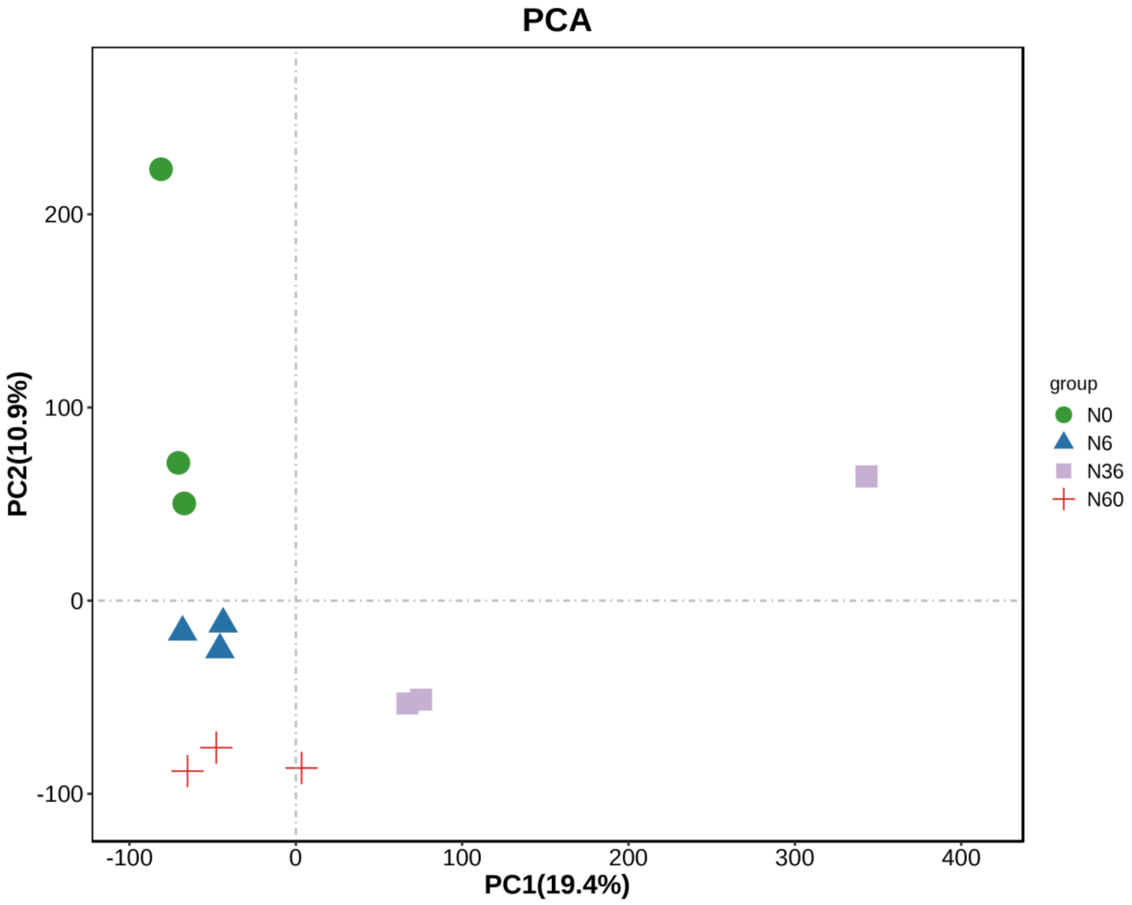

Figure S1. Principal component (PCA) analysis of control (N0) and N treatment (N6, N36, and N60) groups. Total 12 samples as presented in the legend were plotted.

Table S2. Description of the 10 primers designed.

Gene ID	**Forward primer (5′-3′)**	**Reverse primer (5′-3′)**
Cluster-40906.103363	TGAGAAGTAATCCATTGCGAAC	AAGATTCATTCTACGGCCATTC
Cluster-40906.133392	AGAGAATAAAGAGATCAGGCTGGA	GAAATTGTTGTCATGGTAGGGCTA
Cluster-40906.184026	CCAATTTAAAGTAGCAGCAGGGG	GGGGGTTATATGACGAACACGAA
Cluster-40906.58332	GCAAAACTATGGTAAAGGAAACGAC	CTAAAAGCAACTTGAAAAGGGACG
Cluster-40906.99882	ACACATTTTAGAGGATTTATGAGGC	GGATTTCCACTTTGAGGGTTTAT
Cluster-40906.123522	ATGTGGTCATCTGGGATACGCT	CGAACTGATACTTGTTTCACTGCTA
Cluster-40906.92089	TCTCGGTCCCTACTCCCAACA	GCAGTTTCATTGGACGGATTC
Cluster-40906.142461	CTACAAGGTGGGAATACGGGATA	TTGCCGTGTAATGTGGTATGTGT
Cluster-40906.107539	CTATTTACCCAGTCGTTTCCCG	TGTGGAGTTCATCGCAGTCTTT
Cluster-40906.27616	AGGTCGCCAGAACCTTTAGC	CCACACTGAATTCTGGCCCT
NsActin	GGAATCCACGAGACCACCTACA	GATTGATCCTCCGATCCAGACA
